# Effects of High and Low Doses of Folic Acid on the Soluble Receptor Activator of Nuclear Factor-kappa B Ligand/Osteoprotegerin Ratio during Pregnancy

**Published:** 2017-04

**Authors:** Nazila FATHI MAROUFI, Amir GHORBANIHAGHJO, Manizheh SAYYAH MELLI, Maryam VAEZI, Zohreh HEKMATI AZAR MEHRABANI, Maryam BANNAZADEH AMIRKHIZ, Nadereh RASHTCHIZADEH

**Affiliations:** 1. Dept. of Clinical Biochemistry and Laboratory Medicine, Tabriz University of Medical Sciences, Tabriz, Iran; 2. Drug Applied Research Center, Tabriz University of Medical Sciences, Tabriz, Iran; 3. Dept. of Obstetrics and Gynecology, Alzahra Teaching Hospital, Tabriz University of Medical Sciences, Tabriz, Iran

**Keywords:** Folic acid, Osteoprotegerin, Tumor necrosis factorα, Pregnancy

## Abstract

**Background:**

Pregnancy Associated Osteoporosis (PAO) can lead to serious difficulties such as fragility fractures, elongated back pain and height loss in affected women. Soluble Receptor Activator of Nuclear Factor-Kappa B ligand (sRANKL) to Osteoprotegerin (OPG) ratio is chosen as a bone metabolism equation in many bone diseases characterized by bone resorption, such as post-menopausal osteoporosis and would be modified with folic acid supplementation. This study was done to compare the effects of high dose (5mg/day) and low dose (0.5 mg/day) folic acid in the RANKL/OPG ratio and Tumor Necrosis Factorα (TNFα) concentration during pregnancy.

**Methods:**

Forty-five pregnant women who visited the AL-Zahra Hospital, Tabriz Iran, from September 2013 to November 2014 were assigned into two groups in this randomized, double-blind, clinical trial, included women who took 5 mg/day (group1) and who took 0.5 mg/day (Group 2) folic acid supplementation before pregnancy until 36^th^ pregnancy. The biochemical variables in serum of pregnant women were measured before and at the end of the study. The study was registered in the Iranian Registry of Clinical Trials (IRCT) as ID, IRCT2013122315903N1.

**Results:**

OPG levels were significantly higher compared with the baseline value (*P*=0.008), although sRANKL (*P*<0.001), TNFα (*P*=0.005) and sRANKL/OPG ratio (*P*<0.001) reduced significantly with high dose of folic acid supplementation. A significant positive correlation was observed between the decreased RANKL and TNFα levels (r=0.451, *P*=0.031) at the end of study in high dose group.

**Conclusion:**

High dose of folic acid supplementation could decrease bone resorptive biomarkers and may prevent PAO in pregnant women by increasing OPG and decreasing sRANKL and TNFα.

## Introduction

One of the important issues for all women is bone health, particularly during pregnancy (1). Pregnancy is associated with the main changes in calcium metabolism because the developing baby needs plenty of calcium in developing its skeleton, which contains approximately 20–30 gr of calcium and mainly deposited in the latest trimester (1, 2). Adaptive mechanisms involved in protecting a calcium source from pregnant female to the fetus include 1) increased maternal skeleton resorption of calcium and 2) improved intestinal absorption of calcium (3). Enhanced maternal serum free and total 1, 25 hydroxyvitamin D sounds to be responsible for the better intestinal calcium absorption through pregnancy (4, 5). However, increased dietary intake and intestinal absorption are not adequate to provide the calcium required by the fetus (6–8). Therefore, the maternal skeleton still appears to be the potential source of calcium for the fetus (9). Bone turnover in pregnant female increases and Bone Mineral Density (BMD) decreases during gestational period (4, 5–11).

The OPG (Osteoprotegerin)/RANKL (Receptor Activator of Nuclear Factor-Kappa B Ligand)/RANK (Receptor Activator of Nuclear Factor-Kappa B) system was discovered recently and seems to play a fundamental role in bone homeostasis (12). RANKL is a cytokine, which belongs to TNF (Tumor Necrosis Factor) superfamily that regulates the formation and activation of osteoclasts and bone resorption (13). OPG, another cytokine, is capable of protecting bone mass by inhibiting osteoclast differentiation and activation (14). The OPG/RANKL ratio is considered better expose of bone remodeling environment signs. A high ratio of OPG/RANKL indicates bone formation, but a lower favors bone resorption (15). Changes in the OPG/RANKL ratio have been concerned in the pathogenesis of bone diseases characterized by bone resorption, such as post-menopausal osteoporosis (16), and glucocorticoid-induced osteoporosis (17). Maternal plasma OPG concentrations increased in the third trimester of pregnancy, a time when the demand for calcium for fetal bone mineralization is at its maximum level and OPG may protect the maternal skeleton from extreme catabolism (14, 18–20).

The effect of folic acid on bone turnover and bone metabolism has been evaluated in previous studies (21, 22). Furthermore, there is evidence that folic acid supplementation has beneficial effects on bone status (23, 24). Tumor Necrosis Factor α (TNFα) is among the most potent of the osteoclastogenic cytokines that stimulate bone resorption both in vitro and in vivo by increasing the proliferation and differentiation of osteoclast precursors (25).

Thus far, no studies have investigated the effects of TNFα in pregnancy. The maximum rate of bone turnover occurs in the last trimester of the pregnancy, and folic acid supplementation during pregnancy could result in lower bone resorption rates, the present study was carried out to compare the effects of high dose (5mg/day) and low dose (0.5mg/day) folic acid in the RANKL/OPG ratio and TNFα concentration during the pregnancy.

## Materials and Methods

### Subjects

In this randomized, double-blind study, ninety nulliparous women visited the Al-Zahra Hospital, Tabriz, Iran and one auxiliary clinic, Specialized and Sub-specialized Sheykholrais Clinic of Tabriz University, Tabriz, Iran for prenatal tests and planning to be pregnant, were enrolled in this follow-up study from September 2013 to November 2014.

This study protocol was approved by the Ethical Committee of Tabriz University of Medical Sciences (Ethics code: 93/2–4/8) and registered in the Iranian Registry of Clinical Trials (IRCT) and given the ID, IRCT2013122315903N1. Written informed consent was obtained from all participants.

The selection criteria included age between 20–31 yr old, singleton pregnancy, have no history of any disease or medication identified to affect bone metabolism, such as chronic hypertension, diabetes mellitus, chronic renal disease, cigarette smoking or alcohol consumption, early miscarriage and assisted conception. Sixty women consequently became pregnant.

### Blinding

The patients, all persons involved in pregnant women care or treatments, the data collectors, were blind to the treatment distribution. Only the data supervisor of the trial not involved in the treatment or pregnant women care has admission to the treatment distributions.

### Sample collection and analysis

Baseline venous blood samples were collected after overnight fasting at 3-month intervals before conception. After collecting baseline samples, participants received different doses of folic acid (5 mg/d vs. 0.5 mg/d) until 36 wk of pregnancy. The participants were contacted every week and were asked during each visit to declare whether they took folic acid supplementation or not. Second sampling was done in the 36 wk of pregnancy. The participants that had an abortion, multiple pregnancies, usage of calcium and ferrous sulfate during pregnancy and did not complete the follow-up were excluded from the study. Serums were collected after centrifugation (at 3000 RPM) of the samples for 20 min, and then stored at −70 °C until analysis. We used the date of the mother’s last menstrual period (LMP) for calculating Gestation age. Serum OPG and sRANKL concentrations were measured by commercially Enzyme-Linked Immunosorbent Assay (ELISA) kits (Bioassay Technology Laboratory, China, with intra- and inter- assay CV of <10% and <12%, respectively). TNFα was analyzed in serum by ELISA using Immunodiagnostic kit (DIAsource Immuno Assay S.A, Belgium, with intra- and inter- assay CV of 6.6% and 4.5%, respectively).

### Statistical analysis

All of the statistical analyses were performed using SPSS ver. 18 software (SPSS Ins, Chicago, IL). The normality of the distribution of data was tested via the Kolmogorov-Smirnov test. Median (minimum-maximum values), or mean ± SD and their percent when appropriate were used to express the results. For unpaired data, the Mann– Whitney U-test and Independent sample t-test were used, and the Wilcoxon test and a paired Student *t*-test were used for paired data. Evaluation of the correlation was done with Spearman’s test. In all investigated cases *P*-value≤ 0.05, was considered statistically significant.

## Results

Forty-five pregnant women completed follow-up including group 1: twenty-three were treated with 5 mg of folic acid per day and group 2: twenty-two treated with 0.5 mg folic acid per day. Fifteen patients did not complete the study procedure: eight cases had an abortion, two cases had multiple pregnancies and five cases quit taking their supplements. The demographic data and baseline characteristics of two groups are presented in Table 1. No significant differences were observed between two groups at the beginning of the study. The maternal serum levels of OPG, sRANKL and TNFα at the baseline and at the 36^th^ week of pregnancy in both groups have been shown in Table 2. Maternal serum evaluation demonstrated that the levels of OPG in both groups were increased, but only in group 1, there was a statistically significant difference at the 36^th^ week of pregnancy compared to baseline (*P*=0.008).

**Table 1:** Demographic data and Clinical characteristics in in each group (Group1: treat with 5 mg and Group2 with 0. 5 mg folic acid)

**Variable**	**Group1(mean±SD)**	**Group2(mean±SD)**	***P* value**^**2**^
Maternal age (yr)	25.34±3.31	27.04±3.69	0.077
Pre pregnancy Weight (Kg)	68.26±7.75	63.77±5.87	0.060
Height (cm)	164.39±3.22	162.50±3.67	0.071
Pre pregnancy BMI^1^ (kg/m^2^)	25.19±2.09	24.14±1.73	0.125
Interval (days)	49.91±20.29	50.54±19.56	0.910
Ca(mg/dl)	9.01±0.20	8.95±0.21	0.074
P(mg/dl)	3.89±0.15	3.90±.12	0.981
ALP (IU/L)	119.17±36.69	112.42±34.7	0.364
Education			
Under diploma	13(56%)	11(50%)	0.317
High education	10(43%)	11(50%)	

1 BMI; Body Mass Index /

2 Values were obtained by Mann–Whitney U-test.

**Table 2:** Laboratory findings of the participants at baseline and at the end of the study after treatment with high dose and low dose of folic acid (Group1: treat with 5 mg and Group2 with 0.5 mg folic acid)

**Variable**	**Group1(n=23)**	**Group2(n=22)**	***P* value ^5^**
Serum OPG^1^(pg/ml)			
Baseline	340(120–1200)	375(160–1310)	0.488
36^th^week	710(120–1500)	470(130–1400)	
	(*P*=0.008)^4^	(*P*=0.592)^4^	
Serum sRANKL^2^ (pg/ml)			
Baseline	91.6(33.40–198.60)	86.10(39.70–198.60)	0.302
36^th^week	40(20.10–100.20)	76.50(43.70–188.5)	
	(*P*<0.001)^4^	(*P*=0.426)^4^	
Serum TNFα^3^ (pg/ml)			
Baseline	5.30(4.10–10.20)	5.85(4.20–8.90)	0.251
36^th^week	4.80(2.50.–9.00)	6.5(5.00–8.80)	
	(*P*=0.005)^4^	(*P*=0.135)^4^	
Serum sRANKL/OPG			
Baseline	0.27 (0.28–0.17)	0.23(0.25–0.15)	0.555
36^th^week	0.06(0.17–0.05)	0.16(0.34–0.14)	
	(*P*<0.001)^4^	(*P*=0.211)^4^	

1 OPG, Osteoprotegerin;

2 sRANKL, soluble Receptor Activator of Nuclear Factor-kappa B ligand;

3 TNFα, Tumor Necrosis Factorα; Data are expressed as median (minimum–maximum).

4 Differences between before treatment and after treatment with high dose or low dose. (Values were obtained by a Wilcoxon test.) /

5 Baseline high dose group vs. low dose group. (Values were obtained by Mann–Whitney U-test)

The concentration of sRANKL (*P*<0.001) and TNFα (*P*=0.005) significantly decreased in the 36^th^ week of pregnancy compared to baseline in group 1. As shown in Table 2 sRANKL/OPG ratio was decreased in both groups but it was statistically significant only in group 1.

**Fig. 1:**
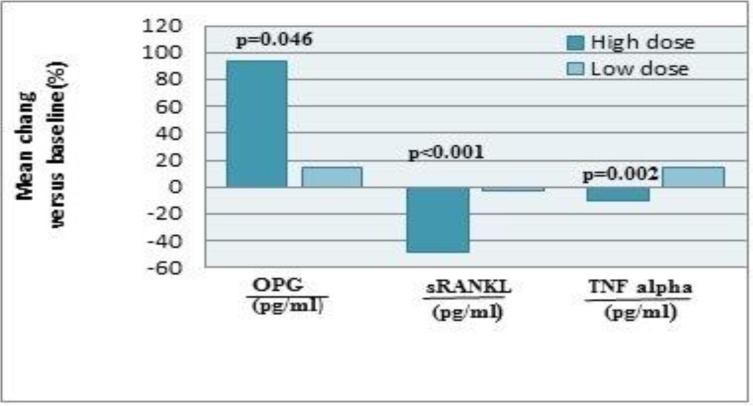
Effect of folic acid supplementation on Percentile variations in serum osteoprotegerin (OPG), soluble receptor activator of nuclear factor-kappa B ligand (sRANKL) and tumor necrosis factor alpha (TNFα) at the end of study between group 1(5mg/day) vs. group 2 (0.5mg/day)(mean)

Bivariate correlation analyses confirmed a positive relationship between the decreased sRANKL (Delta sRANKL) with TNFα (Delta TNFα) (r=0.451, *P*=0.031, Delta sRANKL= (sRANKL at the end of study of High-dose folic acid treatment) — (sRANKL Basal), Delta TNFα= (TNFα at the end of study of High-dose folic acid treatment) — (TNFα Basal)). However, increased OPG levels did not demonstrate an inverse correlation with decreased sRANKL and TNFα levels (r=0.299, *P*=0.166 and r=351, *P*=0.100, respectively) (Table 3). The mean changes in serum OPG, sRANKL and TNFα at the end of the study are shown in Fig. 1.

**Table 3:** Correlation statistics in high dose group (5 mg folic acid)

	**Delta.OPG(pg/ml)**		**Delta.RANKL(pg/ml)**		**Delta.TNFα(pg/ml)**		**Delta.RANKL/OPG**	
	**r**	**P**	**r**	**P**	**r**	**P**	**r**	**P**
Delta.OPG^1^(pg/ml)	_		0.299	0.166	0.351	0.100	0.497	0.016
Delta.sRANKL^2^(pg/ml)	0.299	0.166	_		0.451	0.031*	0.297	0.218
Delta.TNFα^3^(pg/ml)	0.351	0.100	0.451	0.031*	_		0.039	0.859
Delta.RANKL/OPG^4^	0.497	0.016	0.297	0.218	0.039	0.859	_	

* Correlation was significant at the 0.05 level.

1 Delta OPG= (OPG at the end of study of High dose folic acid treatment) — (OPG Basal)

2 Delta sRANKL= (sRANKL at the end of study of High dose folic acid treatment) — (sRANKL Basal)

3 Delta TNFα= (TNFα at the end of study of High dose folic acid treatment) — (TNFα Basal)

4 Delta (RANKL/OPG) = (sRANKL/OPG at the end of study of High dose folic acid treatment) — (sRANKL/OPG Basal

## Discussion

Osteoporosis is a universal problem and affects particularly women (26). PAO seems to be an uncommon problem of pregnancy, but it leads to serious difficulties such as fragility fractures, elongated back pain and height loss in affected women. Definite prevalence of PAO is unknown (27, 28) but to date, about 120 cases have been defined (29). In most cases, PAO develops throughout the first pregnancy and recurrence of PAO with future pregnancies is uncommon, but has been reported to occur (26–28). No standard treatment protocol has been established for the handling of PAO, due to the absence of a convinced etiology (27). PAO causes significant morbidity in the procedure of pain and disability and most patients show various vertebral fractures in the early puerperium and as well as loss of height, which can develop at the end of pregnancy, in the puerperium, or even later (30–34).

OPG referred to as “bone protector” is a cytokine that increases the density and volume of bone tissue by reducing a number of active osteoclasts (14, 35). RANKL is a member of TNF superfamily and its overexpression of soluble RANKL in transgenic mice results in a skeletal phenotype with many similarities to postmenopausal osteoporosis, including reduced BMD, increased bone resorption, cortical porosity and skeletal fragility (15, 34).

OPG can reserve bone loss in models of sex-steroid insufficiency and glucocorticoid-induced osteoporosis, rheumatoid arthritis, multiple myeloma, and metastatic bone disease (12). Since OPG directly counters all RANKL mediated activities through RANK, RANKL/OPG ratio has been an important determinant of bone mass and skeleton integrity. In malignant diseases, such as myeloma, osteolytic bone metastases of prostate and breast cancer, enhanced expression of RANKL by tumor cells and tumor-induced increase of the RANKL/OPG ratio in bone microenvironment can be observed (35). OPG level has progressive increase during pregnancy, with a decrease in serum RANKL (14, 18–20). Our results were in accord with these studies; the pregnant women in both groups showed higher levels of OPG in comparison with baseline, which was significant only in high dose group and significant decrease of sRANKL was observed only in high dose group.

The effect of folic acid was evaluated on bone turnover and bone metabolism (22, 23). The effect of folic acid was evaluated on bone metabolism and turnover during pregnancy (24). Women who took 1 mg of folic acid daily supplement from the beginning of the pregnancy until the delivery time, had significantly higher plasma levels of OPG concentration and lower sRANKL concentration in comparison with women who took the supplements until the end of the second trimester. For the first time, folic acid supplementation during pregnancy could represent low bone resorption rates by higher OPG and lower sRANKL concentrations (24). In our study potential effects of high dose and low dose folic acid supplementation in prevention of bone resorption and PAO during pregnancy is investigated. Our results showed that high dose (5 mg/day) of folic acid significantly decreased the concentration of the serum sRANKL and increased serum OPG and for the first time we showed that sRANKL/OPG ratio decreased in high dose group while low dose of folic acid did not have such effects. Inflammatory cytokines such as IL-1, TNF and M-CSF long associated with osteoclastic bone loss, function by stimulating RANKL production by osteoblast precursors and/or development osteoblasts (36); and/or by decreasing OPG production and/or by up-regulating RANK receptor placement on osteoclast precursors, hence increasing their sensitivity to normal RANKL concentrations (37).

For the first time, we evaluated the effects of high dose and low dose of folic acid on serum TNFα levels. These data showed that in high dose group, TNFα decreased significantly and its decrease had a correlation with decreased sRANKL levels. Moreover, TNFα prompts osteoclastogenesis via the RANKL system, as TNFα up-regulates RANKL mRNA expression (38); and our findings are in agreement with this study. Larger sample size and also measuring new markers of bone resorption, such as serum type 1 collagen C-telopeptide and urine N-telopeptide and bone mineral densitometry will be of great importance to prove the outcomes.

## Conclusion

The complete mechanism of folic acid special effects on the bone metabolism has not yet been absolutely understood; therefore, a series of researches are necessary to reveal unknown features of this process. High dose of folic acid supplementation could decrease bone resorptive biomarkers and may prevent PAO in pregnant women by increasing OPG level and decreasing sRANKL and TNFα levels.

### Ethical Considerations

Ethical issues (Including plagiarism, Informed Consent, misconduct, and/or falsification, double publication and/or submission, etc.) have been completely considered by the authors.
